# Assessing the Quality and Behavior Change Potential of Vaping Cessation Apps: Systematic Search and Assessment

**DOI:** 10.2196/55177

**Published:** 2024-03-15

**Authors:** Fiona McKay, Lilian Chan, Rebecca Cerio, Sandra Rickards, Phillipa Hastings, Kate Reakes, Tracey O'Brien, Matthew Dunn

**Affiliations:** 1 Instutite for Health Transformation, Faculty of Health, Deakin University, Geelong, Victoria, Australia; 2 The School of Health and Social Development, Faculty of Health, Deakin University, Geelong, Victoria, Australia; 3 Cancer Institute of New South Wales, St. Leonards, New South Wales, Australia

**Keywords:** e-cigarettes, quit vaping apps, health apps, behavior change apps, behavior application, behavior, app, application, vaping, smoking, review, vapes, cessation, support, smartphone app, well-being, vape, mobile device, vaping cessation

## Abstract

**Background:**

An increasing number of people are using vapes (e-cigarettes), and with growing evidence of associated harms, there is a need for acceptable cessation support and interventions. Smartphone apps for health and well-being have increased in popularity and use. Limited published literature assesses the potential of apps to support vaping cessation.

**Objective:**

A systematic search of vaping cessation apps currently available in Australia for iOS and Android platforms was conducted. Apps were assessed against established health app assessment tools for quality and behavior change potential.

**Methods:**

A systematic search through the Australian Apple iTunes and Google Play stores was conducted using the search terms “vape”; “vaping”; “e-cigarette”; and “cessation,” “quit,” or “quitting” in May 2023. Only apps that encouraged the cessation of vaping were included. App descriptions were reviewed to determine if they were relevant for inclusion in this study, and relevant apps were downloaded onto the appropriate mobile device for review. The Mobile App Rating Scale (MARS) was used to rate the quality (engagement, functionality, aesthetics, and information) of the apps using an overall score out of 5. The App Behavior Change Scale (ABACUS) was used to assess the behavior change potential of each app using a score out of 21.

**Results:**

An initial search of the app stores yielded 220 Android apps and 124 iOS apps. Screening against the inclusion criteria left 20 iOS apps and 10 Android apps for review. Six apps were available on both operating systems, and these were downloaded, reviewed, and reported separately for each operating system. The average MARS score for all apps assessed in this review was 3.1 (SD 0.41) out of 5. The reviewed apps overall performed well for the MARS elements relating to functionality, such as ease of use and navigation, but had the lowest scores for information-related elements, such as credibility. The number of ABACUS behavior change features per app ranged from 0 to 19 out of 21, with a mean of 8.9 (SD 4.51). The apps commonly included information-related features, such as requesting baseline information. The least common behavior change features were those relating to goal-setting, such as asking about the user’s willingness for behavior change and providing feedback on current actions in comparison to future goals.

**Conclusions:**

The identified vaping cessation apps had moderate levels of quality and some behavior change components. Future vaping cessation apps could benefit from including more features that are known to support behavior change, such as goal-setting, to improve the potential benefit of these apps to support people to stop vaping. As guidelines for vaping cessation continue to be established, future apps need to reference these in their development.

## Introduction

The use of e-cigarettes, also known as vaping, is a growing health issue, particularly among young people [1,2]. There is increasing concern regarding potential links between vaping and lung, heart, and brain damage as e-cigarettes often contain cancer-causing agents, toxins, heavy metals, and very fine particles that can cause adverse health effects [3-5]. There is also concern that vaping is increasing the prevalence of nicotine addiction, and people who vape are three times as likely as those who do not take up tobacco smoking [3]. Young adults and teenagers disproportionally comprise the vaping population [6]. In Australia, 19.8% of people aged 18-24 years used e-cigarettes, compared with 8.9% in the general adult population [7].

Due to the increasing awareness of the adverse effects of vaping, there is a substantial number of people interested in quitting vaping [8]. In this context, the Australian Federal Government has recently legislated significant reforms to limit the accessibility of e-cigarettes [9]. The addictive nature of nicotine in e-cigarettes means that it can be difficult for people to cease vaping, highlighting the need for developing high-quality, easily accessible, and evidence-based cessation supports. There is currently limited evidence on vaping cessation interventions [10]. Research on tobacco smoking cessation has found that mobile phone–based interventions are effective and acceptable smoking cessation support among young adults [10,11]. As nearly all (99%) people aged 18-24 years in Australia have a smartphone [12], this is a potential medium with which to explore vaping cessation support. Preliminary research demonstrates that smartphone apps would be acceptable or preferred as a vaping cessation tool for people aged 14-25 years [13], and a survey among US high school students found that of those seeking vaping cessation support, 18% had used a mobile app or SMS text messaging support [14].

The use of mobile phones to assist with behavior change (also known as mobile health) has become increasingly common, and apps have been developed for a range of health issues, including smoking cessation [15,16], alcohol reduction or cessation [17,18], and increasing physical activity [19]. There has been some research into the effectiveness of health behavior change apps, and several reviews have identified the need for these apps to have greater integration of behavior change theories and techniques [20-22].

Given the recency of vaping as a health issue, there has been scarce research on the use of apps for vaping cessation. A review in 2020 found that most vaping-related apps available on Google Play promoted vaping (they provided instructions on creating e-liquids, finding stores that sell vaping products, etc), and only 3% supported vaping cessation [23]. A subsequent Canadian review identified only 8 apps that were available on both Android and iOS platforms that had been created for vaping cessation [24]. The review assessed the apps for quality and content, and concluded that there were a limited number of apps available for vaping cessation and highlighted a need for more evidence-based practice in the development of future apps.

While the use of apps for self-management of health issues is increasing, assessment of their ability to support people in changing their health behavior is in its nascent stages, and the rapid pace of change in the app market presents a challenge to thoroughly evaluate their effectiveness in changing health behaviors. Assessment tools that appraise apps based on the inclusion of evidence-based behavior change techniques, such as goal-setting and self-monitoring, are a practical way of rapidly assessing the potential of apps to promote health behavior change [25].

As practitioners are increasingly seeking ways to support people to quit vaping, there is a need for information on both the quality and behavior change potential of vaping cessation support apps. This study aims to assess vaping cessation apps available in Australia by conducting a systematic search and using established health app assessment tools to gauge app quality and behavior change potential.

## Methods

### Ethical Considerations

Ethical approval was not required as all data were available in the public domain, and no human participants were involved with this study [26].

### Sample Selection and Inclusion Criteria

**Figure 1.**
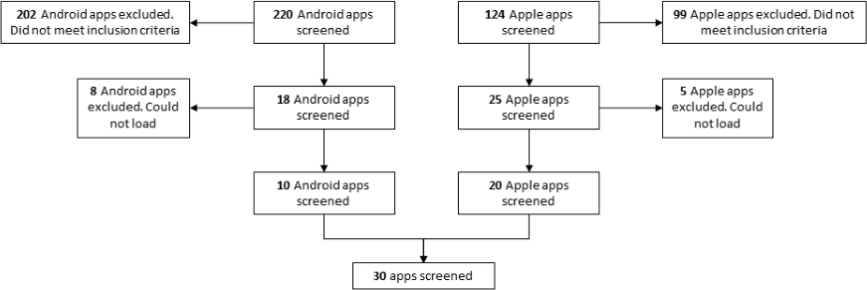
App screening process.

The Australian Apple iTunes and Google Play stores were searched in May 2023 to identify vaping cessation or quitting apps. The Australian app store was searched as the research is based in Australia, and the research team was interested in the apps available in the Australian context. The PRISMA (Preferred Reporting Items for Systematic Reviews and Meta-Analyses) guideline [27] was modified and adapted to guide this review (Figure 1). All apps that were designed to encourage or promote vaping cessation that were available in Australia were included for analysis. Apps that were designed for smoking cessation but contained some vaping cessation components were also included. Apps that sought to promote behavior change but were not specifically related to vaping or e-cigarette cessation were excluded. Search terms were developed to include any vaping apps: “vape”; “vaping”; “e-cigarette”; and “cessation,” “quit,” or “quitting.”

All apps available for download in Australian app stores containing any of the above keywords in either the title or description were downloaded for analysis. App titles and descriptions were read to determine inclusion in the review. The inclusion criteria included apps that were created for vaping cessation and those in the English language. Any apps promoting vaping or e-cigarettes were excluded. While previous studies of apps have inclusion criteria such as an average user rating or recent update [21], given the small number of apps and the recency of vaping cessation as an issue, broad inclusion criteria were used to include all available apps.

All apps were downloaded for use on an iPhone or Samsung Galaxy Android phone. Where an app was available on both Google Play (Android) and Apple iTunes (iOS), the app was downloaded on each device and rated separately on each operating system because features and functionality may vary across the same app when developed for different operating systems.

The title and description of downloaded apps were recorded in an Excel (Microsoft Corporation) spreadsheet and rated. This study used two scales to rate the apps. The first was the Mobile App Rating Scale (MARS) [28] for quality, and the second was the App Behavior Change Scale (ABACUS) [25] to determine the potential for behavior change. The MARS and the ABACUS show good internal consistency and interrater reliability (MARS: α=.90, intraclass correlation coefficient 0.79 [28]; ABACUS: α=.93, intraclass correlation coefficient 0.91 [25]).

### Quality Review and Rating (MARS)

The quality of each app was rated using the MARS [28]. This rating scale examines 19 app elements across four domains: engagement, functionality, aesthetics, and information. Each element was scored using questions on a 5-point ordinal scale. A detailed description of each element and its scoring criteria are provided in the MARS tool [27]. Based on the scores of each element, an aggregated score is calculated for each domain, and an overall app quality mean score (out of 5) is calculated. All apps were reviewed and scored by two authors (FM and MD) who have experience in evaluating health-promoting apps, including those that address addiction and behavior change. Where quality scores differed, the reviewers considered the app together, sought consensus, and determined a final score. The subjective quality rating of the MARS was not included in the analysis due to the subjective nature of this score, in line with commentary from Stoyanov et al [28]. The MARS assessment tool also includes identification of the strategies used by the app (eg, monitoring/tracking, goal-setting, information/education, meditation/mindfulness, and cognitive behavioral therapy) and affiliations of the app (eg, commercial, government, nongovernment organization, or university). These two sections do not contribute to the overall MARS score but are useful to understand the nature and context of the app.

Each app was downloaded and, consistent with other studies [28-30], used for approximately 10 minutes to allow the reviewers to familiarize themselves with the functionality of the app and user experience. Reviewers attempted to use all parts of each app. During the testing, the reviewers noted if the app crashed or if its functions were not accessible. Apps that were not able to be opened or that crashed were removed from the analysis.

### Behavior Change Potential Review and Rating (ABACUS)

The ABACUS [25] comprises 21 items and was used to examine the apps’ potential to support behavior change concerning goal-setting, action-planning, barrier identification, self-monitoring, and feedback. Each app was reviewed and scored by two authors (FM and MD). Each app was first explored by the reviewer to gain familiarity with the app and the interface. Reviewers used all app functions including images, cartoons, videos, record-keeping, calendars, and reminders. A total score out of 21 was calculated by summing the item scores. Where behavior change scores differed, reviewers considered the app together, sought consensus, and determined a final score.

## Results

### Overview of Search

The initial search of the app stores using the specified search terms yielded 220 Android apps and 124 iOS apps (Figure 1). These apps were screened through a review of the title and description; 18 Android apps and 25 iOS apps met the inclusion criteria and were included for assessment and downloaded onto the appropriate device. Eight Android apps and 5 iOS apps could not be downloaded or were no longer available and were excluded from the sample, leaving 10 Android apps and 20 iOS apps included in this review.

### Overview of Included Apps

Most apps that met the inclusion criteria were designed for Apple iOS (n=20), with a smaller number available for the Android operating system (n=10); a minority were available for both operating systems (n=6; see Multimedia Appendix 1 for names of apps). Six apps were created by a commercial organization, 2 by a university, and the remainder (n=22) had an unknown affiliation or developer. Most apps were available for free (n=27); however, of those available for free, most (n=21) had some form of in-app purchase to “enhance” the app experience.

Of the included apps, most used the strategies of allowing the user to monitor/track information (n=25), providing the user with information/education (n=15), providing strategies or tips (n=12), or allowing goal-setting (n=13). A smaller number of apps provided features related to meditation (n=9), cognitive behavioral therapy (n=4), or relaxation (n=2) strategies.

### MARS Assessment for Quality

The average overall MARS score of all reviewed apps was 3.1 (SD 0.41). Individual MARS element mean scores ranged from 4 for the elements “ease of use” and “navigation” to 0 for the element “evidence base,” as no app included any information about scientific trialing or testing of the app. iOS apps and Android apps were of similar quality, both with an overall mean MARS score of 3.1. Across all apps, the functionality domain score was the highest, while the information domain score was the lowest (Table 1).

**Table 1.** Mean app quality calculated using the Mobile Application Rating Scale.

Domain		iOS apps, mean (SD)	Android apps, mean (SD)	Total, mean (SD)
**Engagement score**		2.7 (0.7)	2.6 (0.8)	2.6 (0.7)
	Entertainment	2.4 (0.8)	2.5 (0.9)	2.4 (0.8)
	Interest	2.4 (0.9)	2.7 (1.0)	2.5 (0.9)
	Customization	2.6 (0.8)	2.4 (0.9)	2.6 (0.8)
	Interactivity	2.8 (0.7)	2.2 (1.0)	2.6 (0.9)
	Target group	3.2 (0.6)	3.1 (0.7)	3.1 (0.6)
**Functionality score**		3.9 (0.2)	3.9 (0.5)	3.9 (0.4)
	Performance	3.7 (0.5)	3.7 (0.9)	3.7 (0.6)
	Ease of use	3.9 (0.2)	4.0 (0.5)	4.0 (0.6)
	Navigation	4.0 (0.2)	4.1 (0.4)	4.0 (0.3)
	Gestural design	3.9 (0.2)	4.0 (0.6)	3.9 (0.4)
**Aesthetics score**		3.4 (0.5)	3.6 (0.5)	3.4 (0.5)
	Layout	3.8 (0.3)	3.8 (0.7)	3.8 (0.5)
	Graphics	3.5 (0.5)	3.8 (0.8)	3.6 (0.6)
	Visual appeal	2.8(0.9)	3.3 (0.5)	3.0 (0.8)
**Information score**		2.7 (0.5)	2.5 (0.7)	2.6 (0.5)
	Accuracy of app description	3.1 (0.6)	2.9 (0.6)	3.1 (0.7)
	Goals	2.8 (0.5)	2.3 (1.0)	2.6 (0.7)
	Quality of information	3.1 (0.7)	3.0 (1.1)	3.1 (0.8)
	Quantity of information	3.1 (0.8)	3.0 (1.2)	3.1 (0.9)
	Visual information	2.4 (0.7)	3.1 (1.0)	2.6 (0.9)
	Credibility	1.6 (0.7)	1.6 (0.6)	1.6 (0.7)
	Evidence base	0 (0.0)	0 (0.0)	0 (0.0)
Total objective score		3.1 (0.5)	3.1 (0.4)	3.1 (0.5)

When considering specific MARS elements across all apps, low mean scores were reported for evidence bases (0, as no apps had this element), credibility (1.6, SD 0.67), and entertainment (2.4, SD 0.84). The highest mean scores were obtained for ease of use (4.0, SD 0.33), navigation (4.0, SD 0.29), and gestural design (3.9, SD 0.41). Over half (n=17, 57%) of all apps were rated with an overall MARS score >3.0.

### ABACUS Assessment for Potential to Support Behavior Change

In assessing the apps for behavior change potential, the number of ABACUS behavior change features per app ranged from 0 to 19 out of 21, with a mean of 8.9 (SD 4.51). On average, iOS apps had more behavior change features (9.5, SD 4.6) than Android apps (7.8, SD 4.4). The most common behavior change feature of the 30 apps was the request for baseline information (n=23, 77%), followed by the ability to self-monitor behavior (n=22, 73%) and the ability to personalize or customize the app (n=20, 67%). The least common features were the ability to understand the difference between current action and future goals (n=6, 20%), asking about willingness for behavior change (n=4, 13%), and the ability to export data from the app (n=3, 10%). Table 2 shows the frequencies of the 21 behavior change features evaluated in the apps.

**Table 2.** Behavioral change features identified.

Behavior change feature		iOS apps (n=20),n (%)	Android apps (n=10), n(%)	Total apps (N=30), n(%)
**Knowledge and information**				
	Ability to customize and personalize features	14 (70)	6 (60)	20 (67)
	Consistency with national guidelines or created with expertise	10 (50)	3 (30)	13 (43)
	Request for baseline information	16 (80)	7 (70)	23 (77)
	Instruction on how to perform the behavior	8 (40)	6 (60)	14 (47)
	Information about the consequences of continuing or discontinuing behavior	11 (55)	6 (60)	17 (57)
**Goals and planning**				
	Request for willingness for behavior change	3 (15)	1 (10)	4 (13)
	Setting of goals	8 (40)	4 (40)	12 (40)
	Ability to review goals, update, and change when necessary	6 (30)	3 (30)	9 (30)
**Feedback and monitoring**				
	Ability to quickly and easily understand the difference between current action and future goals	5 (25)	1 (10)	6 (20)
	Ability to allow the user to easily self-monitor behavior	16 (80)	6 (60)	22 (73)
	Ability to share behaviors with others or allow for social comparison	10 (50)	3 (30)	13 (43)
	Ability to give the user feedback—either from a person or automatically	8 (40)	3 (30)	11 (37)
	Ability to export data from app	1 (5)	2 (20)	3 (10)
	Material or social reward or incentive	8 (40)	3 (30)	11 (37)
	General encouragement	11 (55)	5 (50)	16 (53)
	Reminders or prompts or cues for activity	13 (65)	4 (40)	17 (57)
	App encourages positive habit formation	8 (40)	2 (20)	10 (33)
	App allows or encourages practice or rehearsal in addition to daily activities	10 (50)	5 (50)	15 (50)
	Opportunity to plan for barriers	8 (40)	2 (20)	10 (33)
	Assistance with or suggest restructuring the physical or social environment	8 (40)	2 (20)	10 (33)
	Assistance with distraction or avoidance	7 (35)	4 (40)	11 (37)

### Overall Assessment of Individual Apps

The top 5 apps according to their ABACUS and MARS scores are shown in Table 3. While the app with the highest ABACUS score did not have the highest MARS score, the top 5 apps for each operating system were the same, albeit in a different order (see Multimedia Appendix 1 for the ABACUS and MARS scores of all 30 apps included in this review).

**Table 3.** Top apps by App Behavior Change Scale (ABACUS) and Mobile App Rating Scale (MARS) scores.

App name	Operating system	Developer, affiliation	MARS score (out of 5)	ABACUS score (out of 21)	Subjective quality	Costs (up front, in-app purchases)^a^
Quit smoking. Stop vaping app	iOS	Elena Minina, unknown	3.4	19	4	Free up front, no in-app purchases
Quit vaping for good	iOS	Quit Vaping LLC, unknown	3.6	18	4	Free up front, no in-app purchases
Quit Tracker: Stop Smoking	Android	despDev, unknown	3.7	15	2.5	Free up front, Aus $4.99 per feature
QuitSure Quit Smoking Smartly	iOS	Instaquit.org, commercial	3.4	15	3	Free up front, varies Aus $9.99-$39.99
QuitSure Quit Smoking Smartly	Android	QuitSure, commercial	3.8	14	3	Free up front, varies Aus $0.49-$99.99
Kwit Quit smoking for good	iOS	KWIT, unknown	3.8	14	4	Free up front, varies Aus $6.49-$119.99

a A currency exchange rate of Aus $1=US $0.66 is applicable.

## Discussion

### Principal Findings

This review identified 30 vaping cessation apps available in app stores and assessed their quality and potential to support behavior change. The 30 apps were created for either iOS (n=20) or Android (n=10), with 6 being available on both operating systems. On average, the vaping cessation apps performed best in functionality features such as navigation and ease of use, and commonly had behavior change features such as allowing users to self-monitor their behaviors. Areas of deficiency were related to specific behavior change strategies such as comprehensive goal-setting, a lack of evidence of trials or testing, and a lack of transparency in the source of the app and its information.

### Behavior Change Potential

This review goes beyond the existing research [24] by including an assessment of behavior change potential through the use of the ABACUS tool. The mean ABACUS score identified in this review of vaping cessation apps of 8.9 out of 21 is comparable to the mean ABACUS score of apps focused on other health behaviors, such as 7.6 for physical activity apps, 8.0 for apps to reduce alcohol consumption, and 8.7 for apps to improve mental well-being [21]. Notably, the mean ABACUS score for tobacco cessation apps has been reported as 10.2 [21]. As vaping cessation has some similarities with tobacco smoking cessation [31], app developers could look toward smoking cessation apps to identify strategies and features that could be adapted for vaping cessation apps.

The findings of this assessment indicate that there is potential for developers to create vaping cessation apps that include more features known to support behavior change. Goal-setting is an important feature of behavior change interventions [32], but only 12 of the 30 apps included a goal-setting feature. App developers could consider including more comprehensive goal-setting features, such as adding elements that ask the user about their willingness or readiness for behavior change and providing the user with feedback on how their current actions compare to their future goal, such as in the form of a visual graphic. Future apps could also consider the inclusion of additional features that draw on behavior change strategies, such as meditation, cognitive behavioral therapy, and relaxation, particularly as evidence shows that a significant proportion of young people vape to relieve stress or anxiety [33].

### Quality of Apps

The overall quality of vaping cessation apps as assessed by the MARS was moderate, scoring 3.1 out of 5. A review of apps related to other health behaviors identified mean scores ranging from 2.71 for healthy eating apps to 3.26 for apps that aimed to improve mental well-being [21]. One of the areas where vaping cessation apps scored poorly was in credibility, with a mean score of 1.6 compared with a mean of 2.11 that has been identified for health behavior apps overall [21]. It would be valuable for future vaping apps to provide more transparency about their information, development, and funding source. One major challenge is that information and guidelines on vaping cessation are in their infancy and may vary across different countries or jurisdictions. As health organizations begin to publish clinical guidelines for vaping cessation [34,35], it would be beneficial for app developers to consider these in the app development process.

The other key area where apps could be strengthened to provide more potential for behavior change is in the establishment of an evidence base. No reviewed app included evidence of being trialed or tested by the developer or an external party. This is a challenge for health apps broadly as the app landscape changes rapidly, and because app development generally outpaces research and knowledge translation, there is often little time for rigorous research. In practice, developing an evidence base for vaping cessation apps may need to occur in parallel to the promotion and general uptake of these apps, as the need for these apps is already present among people who want to stop vaping, particularly among young people for whom digital cessation support is acceptable [14]. Trialing and evaluation of existing and new vaping cessation apps will allow for continual development and improvement over time, and future apps could benefit from partnerships between app developers, researchers, and health behavior change experts.

### Considerations for Clinicians and Practitioners

Individuals may seek out assistance and advice from their health care providers when considering vaping cessation. While the treatment approaches for tobacco cessation are long established, there is little guidance relating to vaping cessation [36] nor are there evidence-based clinical guidelines on cessation of dual use (the use of both vapes and tobacco cigarettes) [37]. The Canadian Paediatric Society recently provided preliminary clinical guidance on e-cigarette cessation for young people [38]. These guidelines suggest that behavioral therapy, either in combination with or without pharmacotherapy, should be considered when supporting young people in ceasing vaping or e-cigarette use [38]. This is consistent with Australian guidelines [35] and guidelines from the American Heart Association and American College of Cardiology [39] that suggest that practitioners engage in a range of strategies when advising young people on vaping cessation. A recent randomized controlled trial suggested that tailored mobile interventions could be considered an effective tool to support vaping cessation [40]. As such, in their discussions about vaping cessation, practitioners could encourage people interested in vaping cessation to engage in behavior strategies, such as those apps included in this review.

### Considerations for App Developers

Future vaping cessation apps must be developed with an understanding of their target audience and in collaboration with users and clinicians. Users from different age groups may require different features to support their vaping cessation efforts, particularly as they may have different patterns and motivations for vaping [35]. The names and descriptions of some apps included in this review suggest that they were initially developed for tobacco smoking cessation and subsequently adapted for vaping cessation. In Australia, 10.7% of those aged 14 to 17 years are dual users [7], and there are similarities in barriers to and motivations for quitting for both behaviors [31]. Therefore, apps that provide dual support for both health behaviors could be beneficial for some audiences. However, it is important for app developers to create apps with this dual purpose in mind, rather than simply adding vaping cessation to existing smoking cessation app titles.

Finally, it is important to consider the barriers to the uptake of vaping cessation apps. While most of the apps identified in this review were free, many had in-app purchases to access additional features. If a user is unwilling or cannot afford to pay for the additional features, the effectiveness of the app may be compromised. The cost must not be a barrier for people to access health apps [41] and to ensure that people who may not have the ability to pay can access the full level of support for vaping cessation.

### Limitations

While there are important findings presented here, this study presents an analysis of apps available at a point in time (May 2023) and only those available in the Australian app stores—app stores in other countries may have different apps. Although this research provides a reference point for further research into the quality of vaping cessation apps, given the fast-moving nature of this field, app developers may modify and update the features of these apps. While the development of the ABACUS assessment tool included reference to tobacco cessation evidence, the tool has only been studied for validity and reliability for physical activity apps [25]. There are perhaps differences in behavior change techniques relevant to vaping cessation in comparison to physical activity. It is also important to note the potential limitation of reviewing apps that were available on both iOS and Android platforms separately. While this may potentially duplicate the identification of certain features in the pool of available apps, this approach has been designed with careful consideration that recognizes that app features and functionality may vary when developed for different operating systems. Unlike other reviews [21], we did not include the app store star rating in our analysis. Many of the apps identified in the app stores were relatively new and as a result had a small number of reviews, making this an unreliable indicator of quality or usability.

Finally, it is important to note that with these data, we are unable to draw firm conclusions relating to long-term behavior change or provide clinical recommendations. It is also known that apps are sometimes used only for a short duration [42]. This is an area that needs more research attention as we strive to create apps that will be able to assist in improving population health at a low cost.

### Conclusion

This review of vaping cessation apps found that while apps that are currently available performed reasonably in terms of quality, this review suggests that there is room for improvement, particularly in including features that support behavior change. There is a growing interest and need for effective apps to support people to stop vaping, and future vaping cessation apps could be improved by including specific features known to support behavior change, such as goal-setting, meditation, and relaxation activities, and observing the growing body of clinical guidelines for vaping cessation.

## Supplementary material

10.2196/55177 Multimedia Appendix 1 App Behavior Change Scale and Mobile App Rating Scale scores for included apps.

10.2196/55177 Checklist 1 PRISMA (Preferred Reporting Items for Systematic Reviews and Meta-Analyses) checklist.

## Abbreviations

ABACUS: App Behavior Change Scale
MARS: Mobile App Rating Scale
PRISMA: Preferred Reporting Items for Systematic Reviews and Meta-Analyses
