# Supplementary material for: Assessing the Quality and Behavior Change Potential of Vaping Cessation Apps: Systematic Search and Assessment
Source: JMIR Mhealth Uhealth. 2024 Mar 15;12:e55177. doi: 10.2196/55177 (PMC11004626; doi:10.2196/55177)
Supplement: Multimedia Appendix 1 [file mhealth-v12-e55177-s001.docx]

# Multimedia Appendix 1: ABACUS and MARS scores for included apps

| App Name | Operating System | Developer | Affiliations | Upfront cost | In app purchase | ABACUS score | MARS score |
| --- | --- | --- | --- | --- | --- | --- | --- |
| Quit smoking. Stop vaping app | iOS | Elena Minina | Unknown | Free | N | 19 | 3.4 |
| Quit vaping for good | iOS | Quit Vaping LLC | Unknown | Free | N | 18 | 3.6 |
| Quit Tracker: Stop Smoking | Android | despDev | Unknown | Free | Y | 15 | 3.7 |
| Quit Sure | iOS | Quit Sure | Commercial | Free | Y | 15 | 3.4 |
| QuitSure Quit Smoking Smartly | Android | QuitSure | Commercial | Free | Y | 14 | 3.8 |
| Kwit Quit smoking for good | iOS | KWIT | Unknown | Free | Y | 14 | 3.8 |
| Quit Vaping Addiction Calendar | iOS | App Diggity, LLC | Unknown | Free | Y | 13 | 3.6 |
| Quit Smoking NOW - Max Kirsten (paid) | iOS | Life Change Media Ltd | Commercial | 9.99 | N | 12 | 3.2 |
| Quit: Hypnosis to Stop Smoking | iOS | Team Penguin | Unknown | Free | Y | 12 | 3.2 |
| No Vape - CRUSH CRAVINGS | iOS | University of California, San Diego (Business Affairs) | University | Free | N | 10 | 3.5 |
| Escape the Vape | iOS | Woke Enterprises LLC | Unknown | Free | Y | 9 | 2.8 |
| Puff Count | iOS | Steven Cravotta | Unknown | Free | Y | 9 | 3.1 |
| Quit: Hypnosis to Stop Smoking | Android | Team Penguin Studio | Unknown | Free | Y | 9 | 2.9 |
| VapeX - Quit Vaping Today | Android | Behavioral Activation Tech | Unknown | Free | N | 9 | 3.5 |
| QuitVape: Quit Vaping Tracker | iOS | Vasile Curelea | Unknown | Free | Y | 8 | 3.0 |
| Escape the Vape - Quit Juul | Android | Woke Enterprises LLC | Unknown | Free | Y | 8 | 3.1 |
| Quit Guru | iOS | UAB QB CODE | Unknown | Free | Y | 7 | 2.7 |
| stop vaping challenge | iOS | Sherald Sanchez | University | Free | N | 7 | 3.1 |
| VapeX - Quit Vaping Today (paid) | iOS | Behavioral Activation Tech, LLC | Unknown | 4.99 | N | 7 | 2.8 |
| quit easy smoking & vaping | iOS | Harmony Hypnosis Ltd | Unknown | Free | Y | 6 | 2.8 |
| Quit Smoking NOW: Max Kirsten | iOS | Life Change Media Ltd | Commercial | Free | Y | 6 | 2.8 |
| Easy Quit Smoking & Vaping | Android | Harmony Hypnosis Ltd | Commercial | Free | Y | 6 | 3.0 |
| Quit Guru | Android | Quit Guru Ltd. | Unknown | Free | Y | 6 | 3.0 |
| Quitzilla: Bad Habit Tracker | Android | despDev | Unknown | Free | Y | 6 | 2.9 |
| Quitzilla: bad habit tracker | iOS | Andrii Hula | Unknown | Free | Y | 5 | 2.6 |
| Smoke free - Quit Smoking & Vaping Tracker | iOS | CREATIVE TECHNOLOGIES LLC | Unknown | Free | Y | 5 | 2.6 |
| Stop smoking & quit (Quit Smoking Hypnosis by MT) | iOS | ATN Marketing SRL | Commercial | Free | Y | 5 | 2.6 |
| How to Quit Vaping | Android | Not available | Unknown | N/A | N/A | 5 | 2.6 |
| Quit smoking (smoke free - quit smoking) | iOS | 23 Ltd | Unknown | Free | Y | 2 | 2.6 |
| True Quit Stop Smoking App | Android | True Quit | Unknown | Free | N | 0 | 2.1 |
